# Angiogenic Response to Major Lung Resection for Non-Small Cell Lung Cancer with Video-Assisted Thoracic Surgical and Open Access

**DOI:** 10.1100/2012/636754

**Published:** 2012-09-02

**Authors:** Calvin S. H. Ng, Song Wan, Randolph H. L. Wong, Anthony M. H. Ho, Anthony P. C. Yim

**Affiliations:** ^1^Department of Surgery, The Chinese University of Hong Kong, Prince of Wales Hospital, Hong Kong; ^2^Division of Cardiothoracic Surgery, The Chinese University of Hong Kong, Prince of Wales Hospital, Hong Kong; ^3^Department of Anaesthesia and Intensive Care, The Chinese University of Hong Kong, Prince of Wales Hospital, Hong Kong

## Abstract

*Background*. Angiogenic factors following oncological surgery is important in tumor recurrence. Vascular endothelial growth factor (VEGF), angiopoietin 1 (Ang-1), Ang-2, soluble VEGF-receptor 1 (sVEGFR1) and sVEGFR2 may influence angiogenesis. This prospective study examined the influence of open and video-assisted thoracic surgery (VATS) lung resections for early stage non-small cell lung cancer (NSCLC) on postoperative circulating angiogenic factors. *Methods*. Forty-three consecutive patients underwent major lung resection through either VATS (*n* = 23) or Open thoracotomy (*n* = 20) over an 8-month period. Blood samples were collected preoperatively and postoperatively on days (POD) 1 and 3 for enzyme linked immunosorbent assay determination of angiogenic factors. *Results*. Patient demographics were comparable. For all patients undergoing major lung resection, postoperative Ang-1 and sVEGFR2 levels were significantly decreased, while Ang-2 and sVEGFR1 levels markedly increased. No significant peri-operative changes in VEGF levels were observed. Compared with open group, VATS had significantly lower plasma levels of VEGF (VATS 170 ± 93 pg/mL; Open 486 ± 641 pg/mL; *P* = 0.04) and Ang-2 (VATS 2484 ± 1119 pg/mL; Open 3379 ± 1287 pg/mL; *P* = 0.026) on POD3. *Conclusions*. Major lung resection for early stage NSCLC leads to a pro-angiogenic status, with increased Ang-2 and decreased Ang-1 productions. VATS is associated with an attenuated angiogenic response with lower circulating VEGF and Ang-2 levels compared with open. Such differences in angiogenic factors may be important in lung cancer biology and recurrence following surgery.

## 1. Introduction

Surgical resection remains the standard of care for patients with early-stage resectable non-small cell lung cancer (NSCLC). The development of video-assisted thoracic surgery (VATS) has led to a paradigm shift in the approach to lung resection with several studies demonstrating benefits relating to VATS compared with open surgery. VATS is associated with an attenuated inflammatory cytokine response [1], less disturbance of postoperative cellular immunity and immunochemokines [2, 3], shorter hospital stay, less pulmonary [4] and shoulder dysfunction [5], and reduced postoperative pain [1]. These advantages have been attributed to the reduced access trauma related to VATS. 

Nevertheless, some patients develop local or distant recurrent disease even after apparently “curative” lung cancer resection surgery. The mechanisms and circumstances which may favour local recurrence or metastases are highly complex. For disease recurrence, tumor cells need to possess the ability to grow and invade, as well as to be able to evade the host immune system [6]. Angiogenesis, growth of new blood vessel, is an important process to facilitate the growth of tumors beyond a few millimeters in size. This process is regulated at least in part by the balance between numerous pro- and anti angiogenic factors [7]. Interestingly, surgical trauma is known to be associated with altered angiogenic environment. Vascular endothelial growth factor (VEGF) is one of the most potent inducers of angiogenesis, with direct effects on endothelial cell proliferation, migration, and tube formation. In addition, VEGF is known to act as a potent tumor promoter. Release of VEGF following surgery can enhance residual tumor cell growth and potentially encourage metastasis formation [7, 8]. Angiopoietins (Ang) have been shown to destabilize the connections between the endothelium and capillary integrity facilitating the proangiogenic effects of factors such as VEGF [9].

Recently, studies on patients undergoing major colonic resections have shown that abdominal surgical access trauma can cause changes in plasma levels of angiogenic factors, including VEGF [8], Ang [10], and soluble VEGF-receptor (sVEGFR) [11], towards proangiogenic environment in the early postoperative period. Furthermore, when comparing open and laparoscopic colonic resections, the minimal-access approach was associated with an attenuated postoperative angiogenic response [8]. The effects of major lung resection surgery on these plasma angiogenic factors have not been studied. 

The objective of this prospective study was to examine the postoperative circulating levels of angiogenic factors, VEGF, Ang-1, Ang-2, and sVEGFR1 and 2, in patients with early-stage NSCLC undergoing major lung resection by VATS or thoracotomy. 

## 2. Materials and Methods

**Figure 1:**
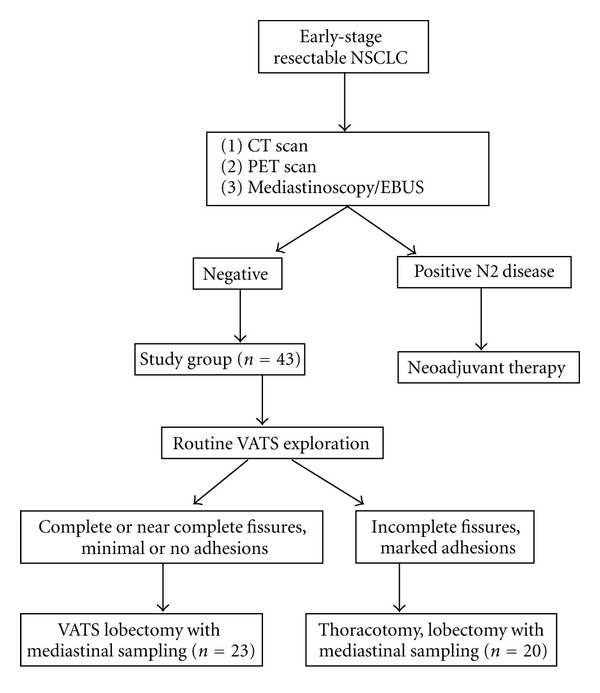
Flow diagram of the 43 patients recruited for the study.

43 consecutive patients with resectable primary NSCLC were recruited over an 8-month period in 2011. Patients with other history of cancer, treated or untreated, were excluded from the study. Ethical approval was given by the research ethics committee of the Chinese University of Hong Kong. Informed consent for the study was obtained from all patients. Standardized preoperative investigations included fiber-optic bronchoscopy, computer tomography of the thorax, and positron emission tomography (PET) scan. At least one invasive staging modality including endobronchial ultrasound (EBUS) and/or mediastinoscopy biopsies was performed. In addition, the results from histology were used for tumor (T), nodal (N), and metastatic (M) staging. VATS resection was carried out whenever it was technically feasible. Patients with fused fissures and marked pleural adhesions were assigned to undergo the conventional posterolateral thoracotomy approach. Major lung resection using individual ligation technique, followed by mediastinal sampling in at least four lymph node stations has been previously described [12] (Figure 1). Both groups of patients received identical anaesthesia with selective one lung ventilation. Our VATS lung resection technique uses 6 to 8 cm nonrib spreading utility minithoracotomy compared with conventional posterolateral thoracotomy. Intraoperative intercostals block with 0.5% bupivacaine (Astra, North Ryde, Australia) was given to both groups of patients at the conclusion of the procedure. Pain control during postoperative days 1 and 2 was achieved by standardized patient-controlled analgesia with meperidine hydrochloride (Antigen Pharmaceuticals Ltd., Roscrea, County Tiperary, Ireland), and subsequently by oral analgesics paracetamol 640 mg and dextropropoxyphene 65 mg four times per day. 

Peripheral venous blood was collected in plain serum tubes (Vacuette, Greiner Bio-One, Kremsmuenster, Austria) prior to anesthetic induction as baseline, and at the same time on postoperative days (PODs) 1 and 3. The sample was allowed to stand for 30 minutes for clotting of blood, followed by centrifugation at 3,000 rpm for 10 minutes at 4°C. The serum collected was stored at −70°C until assay. The concentrations of VEGF, Ang-1, Ang-2, sVEGFR1 and sVEGFR2 were determined by commercially available enzyme-linked immunosorbent assays kits (R&D Systems, Minneapolis, MN, USA), all processed by the same technologist who was blinded to the clinical data.

Fisher's Exact Test was used to detect association of categorical data such as sex and tumor stage. Intragroup differences from the preoperative (baseline) values were detected by the Wilcoxon signed rank test. Mann-Whitney *U* test was used to analyze clinical variables, and any intergroup differences at the various postoperative time points. A two-sided *P-*value of less than 0.05 was considered significant.

## 3. Results

### 3.1. Clinical Findings

The final study consisted of 23 patients in the VATS group and 20 patients in the Open group. No demographic differences were found between the 2 groups (Table 1). None of the patients received mediastinal lymphadenectomy. There was no significant difference between the two groups on the date of resection over the study period, excluding potential effect of possible seasonal variation in the measured parameters. The duration of surgery in the VATS and the Open groups were not significantly different, although there was tendency towards a slightly longer procedure in the latter. The numbers of lymph node stations dissected and sampled were comparable between the groups. No blood transfusion or blood product was required for any patient. There was no perioperative mortality. Two patients in the VATS group had prolonged airleak for 4 days which was self-limiting. In the Open group, one patient developed significant sputum retention requiring bronchoscopic toileting, and one patient developed minor wound infection.

### 3.2. Intragroup Differences in Angiogenic Factors

Compared with the baseline, the levels of VEGF did not change significantly in this early postoperative period (Table 2). Ang-1 levels were significantly lower at POD1 and POD3 following VATS resection. Ang-2 levels rose significantly at POD1 and POD3 following VATS, and at POD3 following Open resection. sVEGFR1 levels rose and sVEGFR2 levels fell significantly at POD1 and POD3 in both groups of patients.

### 3.3. Intergroup Differences in Angiogenic Factors

The baseline values of the measured angiogenic factors were comparable in the 2 groups. The VEGF and Ang-2 levels at POD 3 were significantly higher in the Open group when compared with the VATS group (Table 2). There was a trend towards higher levels of Ang-1 in the Open group.

## 4. Discussion

Although considerable controversy remains, [4, 13] recent observations from nonrandomized trials on VATS resection for stage I lung cancer suggested equivalent or better intermediate to long-term survival compared to conventional thoracotomy [3, 14]. Nevertheless, well-designed randomized trials with longer followup are needed before conclusions can be drawn. The potential survival advantages following VATS major lung resection have led to numerous speculations on the possible mechanisms, including attenuated cytokine-acute phase responses [1] and better preserved immune function leading to improved tumor immunosurveillance [2, 3, 14]. Furthermore, an attenuated proteolytic enzyme response and extracellular matrix degradation following VATS may also be important in reducing the risk of tumor cell invasion and seeding [3]. 

Tumor angiogenesis is known to be an important process which allows tumor growth and metastases to occur [7]. VEGF is one of the most potent inducers of angiogenesis, able to directly induce endothelial cell proliferation, migration, and tube formation. In addition, VEGF can act as a potent tumor promoter. The release of VEGF following surgery could have undesirable effects on residual tumor cells and may enhance tumor growth and metastasis formation. Our study found significantly higher levels of circulating VEGF at POD 3 in the Open group when compared with the VATS group. VEGF plays a key role in wound healing and it is, therefore, logical to assume that plasma VEGF levels following surgery reflect the extent of surgical trauma. Similar findings of higher-postoperative circulating VEGF levels have also been reported when patients undergoing open colectomy were compared with laparoscopic colectomy [8].

Angiopoietin 1 and 2 are proteins found in the blood stream, which compete to bind to endothelial-cell-bound Tie-2 receptor, regulating endothelial cell proliferation, migration, and survival, thereby play an important role in the initiation and regulation of angiogenesis. Ang-1 inhibits apoptosis in endothelial cells and maintains the stability of existing “mature” vasculature [9]. Furthermore, Ang-1 can also inhibit endothelail cell proliferation and invasion into stroma, both of which are VEGF-mediated effects. On the other hand, Ang-2 in most circumstances may destabilize capillary integrity, and through competing with Ang-1 for Tie-2 receptors, decreases Ang-1′s actions [9]. Ang-2 acts to destabilize the connections between the endothelium and the perivascular cells, which enhances the effects of proangiogenic proteins such as VEGF. The current study found increased levels of circulating Ang-2 and decreased levels of Ang-1 in the early postoperative period following major lung resection. These changes in postoperative circulating Ang-1 and Ang-2, which are proangiogenic, are comparable with studies on patients who had undergone major abdominal surgery [10]. Furthermore, we also found that postoperative Ang-2 levels were higher in patients undergoing lung resection by open approach compared with minimal invasive. Such a difference was not found in previous studies comparing open with laparoscopic approach for major abdominal surgery [10]. Implications from differing levels of postoperative Ang-2 following open and minimally invasive lung resection will need further clarification.

The study also found rise in circulating sVEGFR1 levels and a fall in sVEGFR2 levels following major lung resection, although no differences were seen between the two surgical approaches. These changes in sVEGFR have also been observed in patients following major colorectal surgery [11]. sVEGFR are soluble variants of VEGF-receptors (VEGFRs) that are produced by the receptors alternative splicing. The soluble forms have a high affinity for VEGF and sequester free VEGF_165_ in the blood. Since sVEGFR does not have signaling capabilities, sVEGFR1 and sVEGFR2 competitively inhibit VEGF-mediated activation of endothelial cell-bound receptors, and, therefore, to a certain extent discourages angiogenesis [11]. Interestingly, breast and colorectal cancers can express sVEGFR1, with the ratio of sVEGFR1 : VEGF within tumor tissue and in plasma correlating with prognosis, although the exact underlying mechanisms remain unknown [15, 16]. 

Our experience also indicates that in centers where VATS is well established, the operative duration of VATS lung resection is similar or even shorter than that of thoracotomy. Whether such a difference could partially affect the levels of postoperative circulating angiogenic factors remains to be elucidated. 

Although the demographics and pathology of the two groups of patients were comparable in the current prospective study, it suffers from the inherent shortcomings associated with nonrandomized design. However, randomizing patients into VATS or thoracotomy groups is difficult when there is overwhelming evidence to suggest that the former is the superior approach for early lung cancer resection, and that this is well known by our general patient population. Possible bias may arise from the unknown influence of tumor size, staging, presence of pleural adhesions, and surgical complications on angiogenic responses. In terms of the future direction, studies should investigate whether; short-term changes in angiogenic factors can influence tumor behavior, these changes in postoperative circulating angiogenic factors are sustained, and local angiogenic factor responses in the pleural cavity and lung tissue adjacent to the resection margin which may be important in determining local tumor recurrence. In addition, it should be pointed out that early postoperative angiogenic environment following lung resection for cancer may only play a part in the complex interactions between surgical and tumor related factors that influence outcomes.

## 5. Conclusions

Major lung resection for early-stage NSCLC is associated with postoperative proangiogenic environment. Minimally invasive VATS approach resulted in a less angiogenic response in the early postoperative period when compared to the thoracotomy approach. The underlying mechanisms are likely multfactorial, while access trauma may play an important role. The potential impact of postoperative changes in angiogenic factor levels on tumor angiogenesis and recurrence following lung cancer resection warrants further investigation. 

## Figures and Tables

**Table 1:** Clinical data.

Variables	VATS	Open
Patient number	23	20
Age (years)*	63.8 ± 11.1	65.5 ± 7.3
Male/female	15/8	15/5
Smoker	17	16
Tumor diameter (cm)*	3.2 ± 1.6	4 ± 1.9
Tumor histology (Adeno/SCC/other)	13/7/3	14/5/1
Pathological TNM stage		
I	14	12
IIa	5	5
IIb	2	2
IIIa	2	1
Lobectomy/bilobectomy	21/2	19/1
Operative duration (min)	144 ± 39	163 ± 49

*Mean ± SD.

Adeno: adenocarcinoma, SCC: squamous cell carcinoma.

**Table 2:** VEGF, Ang-1, Ang-2, sVEGFR1 and sVEGFR2 levels after VATS and open lung surgery.

	Preop^a^	Day 1^a^	*P*	Day 3^a^	*P*
VEGF (pg/mL)					
VATS	179.2 (100)	174.1 (88)	0.76	170.4 (93)	0.66
Open	246.9 (286)	391.9 (595)	0.72	486.1 (641)	0.17
*P*	NS	NS		**0.04**	
Ang-1 (pg/mL)					
VATS	7504.3 (5944)	3423.3 (2783)	**<0.0001**	3623.9 (2963)	**<0.0001**
Open	7370.7 (6188)	6369.1 (7707)	0.28	7576.6 (8801)	0.47
*P*	NS	NS		NS	
Ang-2 (pg/mL)					
VATS	1932.3 (718)	2581.2 (953)	**0.001**	2484 (1119)	**0.01**
Open	2502 (1236)	3384.2 (1501)	0.07	3378.9 (1287)	**0.026**
*P*	0.052	0.05		**0.026**	
sVEGFR1 (pg/mL)					
VATS	100.1 (22)	140.6 (38)	**<0.0001**	116.7 (45)	**0.042**
Open	85.1 (45)	142.3 (59)	**0.006**	108.4 (52)	**0.015**
*P*	NS	NS		NS	
sVEGFR2 (pg/mL)					
VATS	10469.1 (2220)	9244.2 (2299)	**0.005**	8524.4 (1680)	**<0.0001**
Open	11882 (3016)	9419.4 (2301)	**<0.0001**	8906.3 (2428)	**<0.0001**
*P*	NS	NS		NS	

^a^Mean (SD).
